# Effects of Inorganic Carbon Limitation on the Metabolome of the *Synechocystis* sp. PCC 6803 Mutant Defective in *glnB* Encoding the Central Regulator PII of Cyanobacterial C/N Acclimation

**DOI:** 10.3390/metabo4020232

**Published:** 2014-04-22

**Authors:** Doreen Schwarz, Isabel Orf, Joachim Kopka, Martin Hagemann

**Affiliations:** 1 Universität Rostock, Institut Biowissenschaften, Pflanzenphysiologie, Albert-Einstein-Str. 3, D-18059 Rostock, Germany; E-Mail: doreen.schwarz2@uni-rostock.de; 2 Max-Planck-Institut für Molekulare Pflanzenphysiologie, Am Mühlenberg 1, 14476 Golm, Germany; E-Mails: orf@mpimp-golm.mpg.de (I.O.); kopka@mpimp-golm.mpg.de (J.K.)

**Keywords:** Inorganic carbon, PII-mutant, Primary metabolism, Metabolic fingerprinting, *Synechocystis*

## Abstract

Cyanobacteria are the only prokaryotes performing oxygenic photosynthesis. Non-diazotrophic strains such as the model *Synechocystis* sp. PCC 6803 depend on a balanced uptake and assimilation of inorganic carbon and nitrogen sources. The internal C/N ratio is sensed via the PII protein (GlnB). We analyzed metabolic changes of the Δ*glnB* mutant of *Synechocystis* sp. PCC 6803 under different CO_2_ availability. The identified metabolites provided a snapshot of the central C/N metabolism. Cells of the Δ*glnB* mutant shifted to carbon-limiting conditions, *i.e.* a decreased C/N ratio, showed changes in intermediates of the sugar storage and particularly of the tricarboxylic acid cycle, arginine, and glutamate metabolism. The changes of the metabolome support the notion that the PII protein is primarily regulating the N-metabolism whereas the changes in C-metabolism are probably secondary effects of the PII deletion.

## 1. Introduction

Cyanobacteria are the only oxygenic phototrophs among prokaryotes. During the photosynthetic carbon assimilation they convert inorganic carbon (C_i_) into organic molecules [1]. CO_2_ is used as main carbon source and fixed via ribulose-1,5-bisphosphate carboxylase/oxygenase (Rubisco) in the Calvin-Benson cycle, whereas bicarbonate can additionally be incorporated into oxaloacetate via phosphoenolpyruvate (PEP) carboxylase. Not only C_i_ but also appropriate inorganic nitrogen sources are necessary for the production of cyanobacterial biomass. The variety of possible N-sources is much wider than that of C-sources. Many cyanobacterial strains are able to fix atmospheric N_2_. These diazotrophic strains are independent from external, combined nitrogen sources. However, even these strains prefer the utilization of NH_4_^+^ or NO_3_^−^ as energetically cheaper inorganic N-sources over N_2_-fixation. Thus, NH_4_^+^ or NO_3_^−^ can be used by almost all cyanobacterial strains to synthesize amino acids, mainly glutamate via the glutamine synthetase/glutamine 2-oxoglutarate (2OG) aminotransferase (GS/GOGAT) pathway. Many cyanobacterial strains also are able to use alternative organic N-compounds such as urea, arginine and some others [2].

In the natural environment, the amount and nature of C- as well as N-sources shows great variability. The acclimation toward these changes is well understood for unicellular, non-diazotrophic cyanobacterial model strains such as *Synechocystis* sp. PCC 6803 (hereafter *Synechocystis*). To cope with low and varying C_i_ amounts, *Synechocystis* as well as all other extent cyanobacteria evolved an efficient C_i_ concentrating mechanism (CCM) that combines active transporters for C_i_ and the compartmentalization of Rubisco together with carbonic anhydrase into the bacterial compartment carboxysome. This mechanism allows the accumulation of bicarbonate inside the cell and its conversion into CO_2_ near Rubisco inside the carboxysome [3,4]. Many genes coding for CCM components are up-regulated under C_i_-limiting conditions [5]. Two transcriptional factors, CmpR [6] and NdhR (CcmR) [7,8], are known to be involved in this process. Recently, it has been shown that the promoter binding of these LysR-type transcriptional factors is modified by the association of specific metabolites such as 2-phosphoglycolate (2PG) and ribulose-1,5-bisphosphate in the case of the activator protein CmpR [9] or 2OG and NADP^+^ in the case of the repressor protein NdhR [10]. The altered gene expression results in an increased C_i_ affinity and a coordinated change in the metabolome [11,12]. The acclimation to low nitrogen availability also includes the activation of multiple transporters for combined N-sources as well as enzymes involved in specific routes of the N-assimilation. The transcriptional factor NtcA has been shown to be responsible for the coordinated regulation of corresponding low-N-responsive genes [2,13]. The promoter-binding activity of the activator protein NtcA also is stimulated by the association with the low molecular co-activator molecule 2OG [14,15,16].

**Figure 1:**
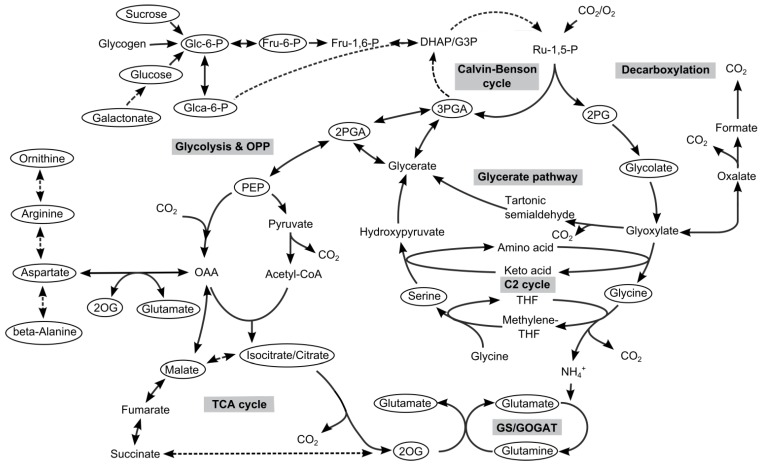
Primary C- and N-metabolism of cyanobacteria. Metabolites highlighted by circles were determined in our metabolome profiling analysis.

Abbreviations: Glc-6-P, glucose-6-phosphate; Fru-6-P, fructose-6-phosphate; Glca-6-P, gluconate-6-phosphate; DHAP, dihydroxyacetone phosphate; G3P, glyceraldehyde-3-phosphate; 3PGA, 3-phosphoglycerate; 2PGA 2-phosphoglycerate; PEP, phosphoenolpyruvate, 2PG, 2-phosphoglycolate; 2OG, 2-oxoglutarate; OAA, oxaloacetate.

However, the response toward different C- and N-levels must be coordinated to obtain a balance C/N ratio. The metabolite 2OG is believed to act as the central sensing molecule for the cellular C/N status, because 2OG is the direct precursor for the GS/GOGAT pathway, thus it connects C- and N-metabolism in cyanobacteria (see Figure 1). In addition to the 2OG-mediated regulation of the transcriptional factors NdhR and NtcA, 2OG also affects the structure and activity of another regulator protein, called PII [17]. PII proteins are found in all three kingdoms of life. In most cyanobacteria including *Synechocystis* it is encoded by the *glnB* gene and forms homotrimeric proteins, which beside 2OG can bind ADP or ATP. Furthermore, cyanobacterial PII proteins can be phosphorylated at a defined serine residue depending on the 2OG or ADP/ATP binding to the PII trimer [18,19]. Thus, it is believed that PII proteins monitor and regulate especially the N-metabolism depending on the C/N ratio and the energy status of the cell. This regulation is realized via the binding of PII to different effector proteins such as the key enzyme for arginine biosynthesis or the transporters for inorganic N-sources [17]. Of crucial importance is the control of PII over the transcriptional regulator NtcA that is realized via the PipX protein. The PipX protein is released from the PII-2OG complex formed under low N/high C conditions. This release causes the activation of NtcA due to the binding of NtcA to the co-activator molecules 2OG and PipX. This transcriptional active complex exists under N-limiting conditions and guarantees the activation of N-assimilatory genes. Under N-replete conditions, 2OG concentrations are low and PipX favors binding to PII rather than to NtcA. Thus, transcriptional activation by NtcA is abolished in the presence of sufficient N [20,21,22].

Previously, we showed that mutation of *glnB* in another model cyanobacterium *Synechococcus elongatus* PCC 7942 had only minor impact on the metabolome and mostly affected the down-regulation of N-assimilatory genes under C_i_-limiting conditions (increase N/C ratio), whereas the up-regulation of Ci-specific genes was mostly non-affected [23]. Here we analyzed how the absence of PII affects the changes in the metabolome under varying C_i_-levels in cells of *Synechocystis*.

## 2. Results and Discussion

### 2.1. Physiological Characterization

**Figure 2:**
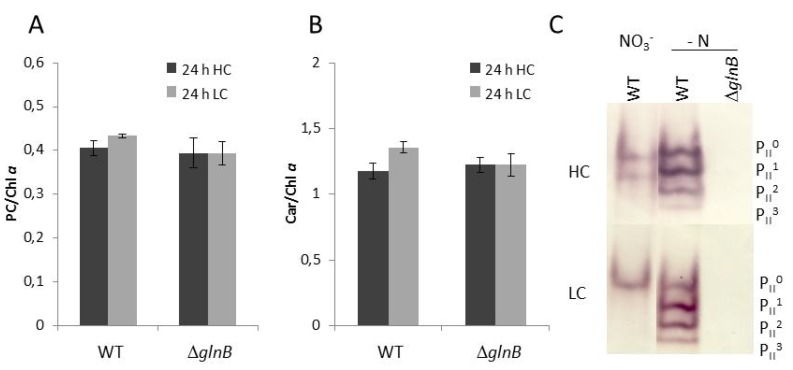
Pigmentation and PII phosphorylation of cells of the wild type (WT) and the PII mutant Δ*glnB* of *Synechocystis* sp. PCC 6803 grown under high CO_2_ conditions (5%, HC) or low CO_2_ conditions (0.038%, LC). **(A)** Phycocyanin (PC) relative to chlorophyll a (Chl*a*). (**B**) Carotenoids (Car) relative to chlorophyll a (Chl*a*). (**C**) Detection of the different phosphorylated forms of the PII trimer in the WT and the Δ*glnB* in cells grown under HC or LC in standard BG11 medium (NO_3_^−^) or in BG11 medium without combined nitrogen source (- N). The different phosphorylation states of the PII trimer are visualized by immuno-blotting after separation of 10 µg of soluble proteins by native PAGE (P_II_^0^: dephosphorylated PII protein, P_II_^3^: complete phosphorylation of all three PII monomers).

Under high CO_2_ conditions (HC, 5% CO_2_), the growth of the mutant Δ*glnB* was about two times slower than that of the wild type (WT) (data not shown). The transfer to low CO_2_ conditions (LC, 0.038% CO_2_) diminished the growth of the mutant and that of the WT to about the same extent. Our observation of decreased growth of the *Synechocystis* PII mutant at HC as well as LC corresponds to the previously published growth data of this mutant [24]. The PII mutant of *Anabaena* sp. PCC 7120 also showed only 50% of the growth rate of WT cells regardless if the cells were grown with nitrate or under N_2_-fixing conditions [25]. Moreover, both strains showed differences in color, *i.e.* the pigmentation of the *Synechocystis* PII mutant looked more yellowish under HC conditions. This different appearance is due to decreased phycocyanin content (phycocyanin in HC cells of WT 1.85 ± 0.08 and PII mutant 1.35 ± 0.07 µmol L^−1^ OD750^−1^ (n = 6)), whereas the pigment ratios showed only slight changes (Figure 2). This difference became smaller under LC conditions. Decreased chlorophyll *a* and especially phycocyanin contents also have been reported for PII mutant of *S. elongatus* PCC 7942 [26].

We also checked how the different amounts of CO_2_ as well as nitrogen limitation affect the phosphorylation of the PII protein in WT cells. As shown previously [11,18], the PII phosphorylation state increased under N-limiting compared to nitrate-containing conditions in *Synechocystis*, whereas CO_2_-limiting conditions (LC) decreased the PII phosphorylation state. The PII protein became completely dephosphorylated under LC conditions in nitrate grown cells (Figure 2). The immuno-blot with protein extracts from the mutant Δ*glnB* showed no signals confirming the complete absence of PII protein in these cells.

### 2.2. Metabolome Analysis

Our metabolome data set comprised 693 mass features. As discussed recently [27], only a rather small fraction could be assigned to 79 defined metabolites (Supplemental Table 1). The identified metabolites allowed a good characterization of the primary carbon and associated nitrogen metabolism (Figure 1). The mutation of *glnB* had an important effect on the amounts and the pattern of identified metabolites. About 30% of these metabolites showed significant changes in cells of mutant Δ*glnB* compared to the WT.

#### 2.2.1. Low Carbon Signature Metabolites

Previous investigations [11,12,23] on LC-shift-induced alterations in the metabolome of cyanobacteria revealed a general signature of metabolic changes. A major part of carbon is used for carbohydrate biosynthesis and glycogen storage under HC conditions. In contrast, LC conditions favor the export of carbon out of the Calvin-Benson cycle towards the glycolytic pathway, which leads to a LC-characteristic accumulation of 2-phosphoglycerate (2PGA) and PEP. In addition, the shift into CO_2_-limiting conditions activates at least transiently the photorespiratory 2-phosphoglycolate (2PG) metabolism leading to a transient accumulation of 2PG, glycolate, and glycine.

**Figure 3:**
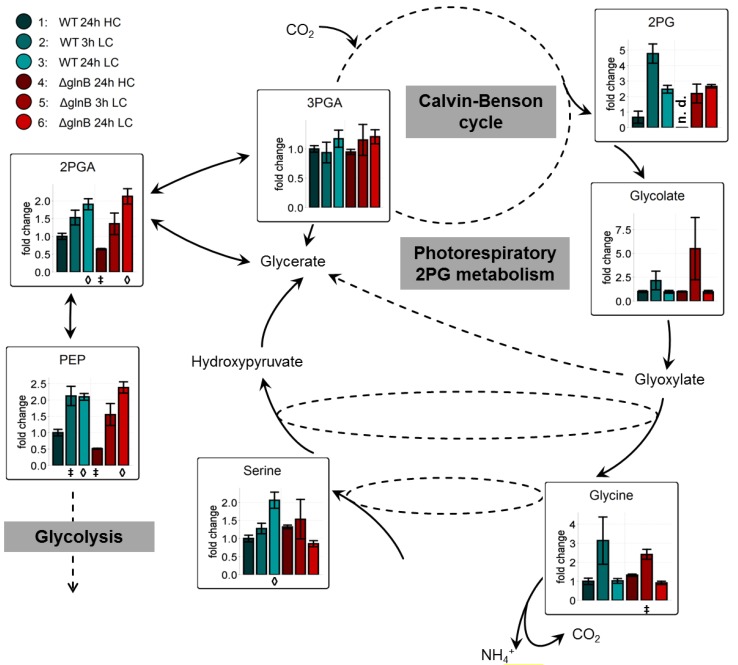
Changes of the accumulation of low carbon signature metabolites in cells of the wild type (WT) and the PII mutant (Δ*glnB*) of *Synechocystis* sp. PCC 6803 after shifts from high CO_2_ (5%, HC) to low CO_2_ (0.038%, LC) conditions. Colored bars correspond to averaged values of three biological replicates measured by at least two technical replicates. Relative changes of metabolite pool sizes are represented by x-fold factors calculated relative to WT 24h HC. Error bars represent the standard errors (n.d. not detectable). Student’s T-test was applied to identify significant changes compared to WT 24 h HC.

‡: *p*-value ≤ 0.05, ◊: *p*-value ≤ 0.0). Abbreviations see Figure 1.

The mutation of *glnB* had a rather minor impact on this general changes. In our present data set, cells of the WT and the mutant Δ*glnB* showed almost the same relative increase in 2PGA, PEP, and 2PG in extracts from cells 24 h after the LC shift. Both strains also transiently accumulated increased amounts of the 2PG metabolism intermediates glycolate and glycine (Figure 3). Only the intermediate serine behaved differently in WT and mutant cells. Its amount increased in LC-shifted cells of the WT, whereas it showed a slightly lower level in LC compared to HC cells of the mutant Δ*glnB*. Collectively, these findings imply that PII is only little involved in the redirecting of carbon fluxes in *Synechocystis* under differing CO_2_ availability.

#### 2.2.2. PII Deletion Increases Carbohydrate Consumption at Low CO_2_ Conditions

**Figure 4:**
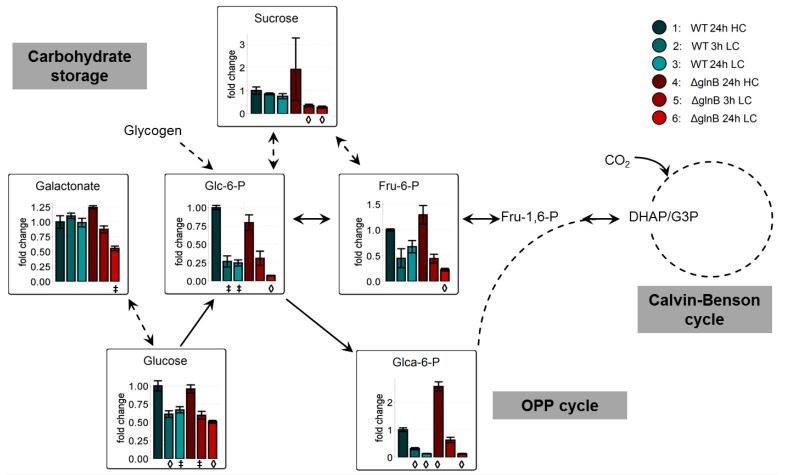
Changes of carbohydrate metabolism in cells of the wild type (WT) compared to the PII mutant (Δ*glnB*) of *Synechocystis* sp. PCC 6803 after shift from high CO_2_ (5%, HC) to low CO_2_ (0.038%, LC) conditions. Colored bars correspond to averaged x-fold values of three biological replicates measured by at least two technical replicates. Factors are calculated relative to WT 24h HC. Error bars represent standard error (n.d. = not detectable). Student’s T-test was applied to identify significant changes compared to WT 24 h HC.

‡: *p*-value ≤ 0.05, ◊: *p*-value ≤ 0.01). Abbreviations see Figure 1.

Another characteristic feature of LC-acclimated cyanobacterial cells is the decrease in the storage of glycogen and the pools of soluble carbohydrates [11,12]. This trend was confirmed under the experimental conditions of this study (Figure 4). Glucose and some phosphorylated sugars showed clearly lowered values in cells of the WT as well as the mutant Δ*glnB* when shifted from HC into LC conditions. In many cases, the decrease seemed to be more pronounced in cells of the mutant Δ*glnB* after 24 h growth at LC. For example, the amounts of fructose-6-phosphate (Fru-6-P) and glucose-6-phosphate (Glc-6-P) showed about half of the level of WT cells at this time point (Figure 4). Obviously, the cells of mutant Δ*glnB* used more of these Calvin-Benson cycle and Oxidative Pentose-Phosphate (OPP) cycle intermediates for biosynthetic purposes than WT cells. Alternatively, the HC grown cells of the PII mutant might have stored less glycogen than WT under HC, hence the storage is earlier consumed after LC shift leading to lowered final levels of this intermediates. Decreased glycogen accumulation has been shown for nitrate-grown cells of the *S. elongatus* PCC 7942 PII mutant [26].

#### 2.2.3. PII Deletion Strongly Decreases Ammonia Assimilation via GS/GOGAT

**Figure 5:**
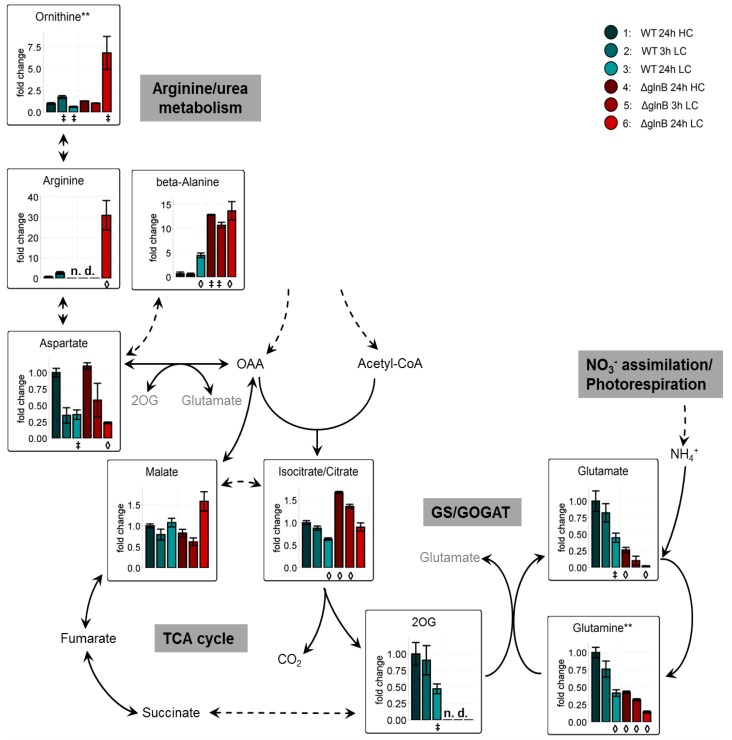
Changes of primary N-metabolism and its precursors in cells of the wild type (WT) compared to the PII mutant (Δ*glnB*) of *Synechocystis* sp. PCC 6803 after shift from high CO_2_ (5%, HC) to low CO_2_ (0.038%, LC) conditions. Colored bars correspond to averaged x-fold values of three biological replicates measured by at least two technical replicates. Factors are calculated relative to WT 24h HC. Error bars represent standard error (n.d. = not detectable). Student’s T-test was applied to identify significant changes compared to WT 24 h HC.

‡: *p*-value ≤ 0.05, ◊: *p*-value ≤ 0.01. Abbreviations see Figure 1. ** Due to the chemical derivatization required for the GC-MS based profiling of primary metabolites the glutamine measurements represent the sum of endogenous pyroglutamate, glutamine, and minor contributions of glutamate. The ornithine measurements represent endogenous ornithine, citrulline, and arginine. The arginine measurement is taken from a specific chemical derivative.

Compared to the missing or rather slight changes in the values of metabolites involved in Calvin-Benson cycle, glycolysis, photorespiration, OPP cycle, and carbohydrate metabolism, we found dramatic changes in metabolites connected to GS/GOGAT and N-detoxification between WT and Δ*glnB* cells. The known decrease in glutamate and glutamine levels after LC shifts in WT cells was correlated with a decreased expression of the corresponding genes in LC-shifted cells [5]. Similarly, *glnA* expression also became decreased in WT cells of *S. elongatus* PCC 7942 [23]. However, the decrease in glutamate and glutamine levels was much more pronounced in cells of the mutant Δ*glnB*. Already at HC, the contents of these amino acids were only 30% compared to WT, but under LC conditions the pools of these amino acids approximated the detection limit (Figure 5). Their decrease might be at least partly related to a reduced activity of GS/GOGAT. Decreased GS activity has been shown in the PII mutant of *Anabaena* sp. PCC 7120 [25]. The decrease of *glnA* expression under LC conditions was about two-times higher in the Δ*glnB* mutant of *S. elongatus* PCC 7949 compared to WT [23]. However, for the *Synechocystis* PII mutant rather increased GS activities and *glnA* mRNA levels have been reported, whereas the nitrite reductase was somewhat lower compared to WT [28]. Thus, beside regulatory effects on GS amount and activity, this decrease is probably more directly related to the strong decrease in the 2OG level in the *glnB* of *Synechocystis*. WT cells showed a stable 2OG pool at 3 h LC and a slight decrease at longer times at LC. In contrast, 2OG was below the detection threshold in extracts from the mutant Δ*glnB* regardless of the growth conditions (Figure 5). This finding contrasts the behavior of LC-shifted *S. elongatus* PCC 7942 WT and Δ*glnB* cells, which showed rather an increase in 2OG than a decrease under LC conditions [23]. It has been shown that the Δ*glnB* mutants of *S. elongatus* PCC 7942 contained a point-mutated, inactive gene encoding for the PipX protein [29]. In contrast, the *Synechocystis* Δ*glnB* mutant used in the current study shows no alterations in the DNA sequence of the *pipX* gene (*ssl0105*) including 250 bp of the up- and downstream regions (personal communication of the Forchhammer group, University Tübingen, Germany). Thus, the different behavior of the *S. elongatus* PCC 7942 and *Synechocystis* Δ*glnB* mutants could be based on this genotypic difference. The lowered 2OG level in cells of the *Synechocystis* PII mutant is unlikely to be explained by a limited flux of carbon through glycolysis or into the TCA cycle, because glycolytic intermediates such as 2PGA and PEP did not show significant differences between cells of the WT and the Δ*glnB* mutant (see Figure 3).

Interestingly isocitrate, the precursor of 2OG synthesis, showed almost a twofold increase in cells of the mutant Δ*glnB* compared to WT under all growth conditions. One possible explanation could be a decreased activity of the isocitrate dehydrogenase (IDH) activity in the mutant leading to an accumulation of isocitrate. It has been found that the expression of the *icd* gene coding for IDH is about twofold decreased in LC-shifted *Synechocystis* cells [5]. Correspondingly, an increased expression of *icd* was reported for N-limited cyanobacteria including *Synechocystis* [30]. Similarly decreased *icd* mRNA levels were found in LC-treated cells of *S. elongatus* PCC 7942, but the expression was not further reduced in the corresponding mutant Δ*glnB* [23]. The transcriptional regulation fits to the main role of IDH to synthesize the precursor 2OG for glutamate synthesis, which is decreasingly needed under lowered N/C conditions. In addition to the expression level, the IDH activity is known to be regulated on biochemical level in cyanobacterial cells. For example, the purified IDH from *Synechocystis* showed inhibition by 2OG and NADPH_2_ [31]. Whereas the 2OG inhibition is not relevant to explain the increased isocitrate levels in cells of the mutant Δ*glnB*, higher amounts of NADPH_2_ can be assumed in mutant cells due to the lowered N-assimilation, one major sink for redox in cyanobacterial cells. Whether or not IDH activity also is directly redox-regulated is a matter of discussion. IDH of *S. elongatus* PCC 7942 showed no sign of redox regulation [32], whereas IDH from *Anabaena cylindrica* could be activated by spinach thioredoxin [33]. Recently, the IDH was found to be among the redox-regulated proteins upon nitrogen starvation in *Prochlorococcus* sp. SS120 [34]. Unfortunately, direct redox-effects were not investigated with the purified *Synechocystis* IDH [31]. However, it is likely that the redox state in cells of the mutant Δ*glnB* is changed leading to decreased IDH activity and thus contributing to the decreased isocitrate consumption. A lack of carbon flux in the direction to amino acids such as glutamate via 2OG could also explain the increase in malic acid in long-term LC cultivated cells of the mutant Δ*glnB* (Figure 5). Like glutamate, the amount of aspartate drops to low levels in cells of the mutant Δ*glnB* 24 h after the shift to LC conditions. Possibly, then the oxaloacetate is used to a lesser extent for aspartate biosynthesis leading to an enhanced C-flux toward malate.

Dramatic changes also occurred in the levels of arginine and ornithine. Our analysis indicated that these two N-containing metabolites increased 30fold (arginine) and 7fold (ornithine) in cells of the mutant Δ*glnB* after 24 h in LC. The increase in arginine could be related to the diminished cyanophycin accumulation in the *Synechocystis* PII mutant [35]. It has been reported that when ammonia was added to N-limited *Synechocystis* WT cells high amounts of cyanophycin were accumulated, whereas the PII mutant did not show this typical elevated cyanophycin levels. However, the addition of arginine also induced the cyanophycin accumulation in the PII mutant albeit to only 30% of the WT level [35]. These differences were attributed to the changed activity of the N-acetyl-L-glutamate kinase (NAGK), the key enzyme for arginine biosynthesis, which is known to be directly controlled by the PII protein [17]. This enzyme is less active in the PII mutant [35], since the PII-induced reduction of the feedback inhibition of NAGK by arginine is abolished in the mutant. Thus, the *de novo* synthesis of arginine should be lowered in the mutant. Probably, the accumulation of arginine as well as ornithine is rather a sign of a deregulated N assimilation. PII is known to down-regulate nitrate uptake and assimilation under conditions of excess nitrate in the medium [17], *i.e.*, LC conditions leading to a decreased C/N ratio. Gene expression analysis with a *S. elongatus* 7942 Δ*glnB* mutant indicated that PII protein was important to allow NtcA the nitrogen-deprivation regulated activation (such as *glnA*) or repression (such as *rbcS*) of genes, since these responses were almost completely abolished in in a *glnB* mutant as well as in the *ntcA* defective strain [36]. Accordingly, the *Synechocystis* mutant Δ*glnB* has been shown to excrete nitrite due to an uncontrolled uptake of excess nitrate [37]. Clearly increased nitrate reductase activity also was found in nitrate-grown cells of the *glnB* mutant of *Anabaena* sp. PCC 7120 and the excess of combined nitrogen compounds was excreted here as ammonia instead of nitrite from the mutant cells [25].

Strikingly, the second precursor of cyanophycin, aspartate shows an inverse behavior to arginine in the *Synechocystis* mutant Δ*glnB* (Figure 5). The aspartate response resembles the response-pattern of carbohydrates, *i.e.*, carbohydrates are decreased under LC conditions and deplete further in cells of the mutant Δ*glnB*. Additional to cyanophycin and protein synthesis, aspartate can be consumed by the reaction of aspartate decarboxylase especially under conditions of deregulated N assimilation. This reaction generates beta-alanine. Most probably, the decreased aspartate pool is connected to the more than 10fold accumulation of beta-alanine under LC conditions (Figure 5). This pathway could be used to detoxify excess nitrogen in cells of the *Synechocystis glnB* mutant. In the presence of an intact PII protein, N assimilation is down-regulated under LC conditions. Accordingly, WT cells that were exposed for 24 h to LC conditions decrease expression of many N-regulated genes [5,23] and accumulate much lower amounts of beta-alanine. Together our observations support the hypothesis that beta-alanine generated via aspartate may represent an indicator of an N/C imbalance. 

## 3. Experimental Section

### 3.1. Strains and Culture Conditions

Axenic cells of the *Synechocystis* sp. PCC 6803 wild type and the mutant defective in the PII protein Δ*glnB*::Sp [24] were routinely grown in medium BG11 [38] at pH 8, 29 °C and continuous light of 120 µmol photons m^−2^·s^−1^. Mutant cells were cultivated in BG11 medium supplemented with 20 µg mL^−1^ spectinomycin (Sp). Cells were pre-cultured under high CO_2_ (HC) conditions by sparking the cultures with air enriched with 5% CO_2_. Low carbon (LC) conditions were induced by transferring cells into BG11 medium pH 7 and sparking with ambient air (0.038% CO_2_). To test PII phosphorylation under N-limiting conditions, cells were grown for 16 h in nitrate-free BG11 medium under HC or LC conditions. Contamination by heterotrophic bacteria was checked by spreading of 0.2 ml culture on LB-plates and incubating them for 48 h at 30 °C.

### 3.2. Metabolome Analysis

Cells grown in liquid media under standard HC or LC conditions were harvested by fast filtration in the light and immediately frozen in liquid nitrogen as described previously [23]. All experiments were repeated using three independent cell cultures. From each culture at least two replicates were analyzed. Metabolites were determined using a metabolite-targeted profiling method, and a quantitatively standardized and calibrated variant of the previously established gas chromatography electron ionization time-of-flight MS (GC-EI-TOF-MS) profiling analysis [11,39]. Relative amounts of metabolites were estimated by adding a defined amount of an internal standard, D-^13^C_6_-sorbitol (CAS 121067-66-1) and L-2,3,3,3-d_4_-alanine- (CAS 18806-29-6) (Sigma-Aldrich Chemie GmbH, Munich, Germany), to the cell material. The amounts were normalized to biomass using the optical density at 750 nm (OD_750_) of each sample [11,12]. Guidelines for manually supervised metabolite identification were the presence of at least 3 specific mass fragments per compound and a retention index deviation < 1.0% [40]. Metabolites were routinely assessed by relative changes expressed as response ratios, *i.e.* x-fold factors of LC-shifted cells in comparison to the condition WT 24 h HC.

### 3.3. Statistical Analyses

Statistical testing, e.g. Student’s T-test was performed using log_10_-transformed response ratios. Statistical assessments were performed using the Microsoft-Excel 2010 program.

### 3.4. Physiological Characterization

The growth of the cells was monitored by measurements of OD_750_. Pigment concentrations were calculated after evaluation of *in vivo* absorption spectra as described by reference [23]. To monitor the PII protein phosphorylation status, soluble proteins were isolated from 50 mL of cells. Equal amounts of proteins (10 µg) were separated in 15% acrylamide gels under native conditions and subsequently blotted onto nylon membranes. The PII protein bands were detected by a PII-specific antibody (received from K. Forchhammer, University of Tübingen, Tübingen, Germany). The immune-blotting method is described in more detail by reference [11].

## 4. Conclusions

The comparison of metabolic changes in LC-shifted cells of the *Synechocystis* WT and the Δ*glnB* mutant supported the notion that the PII protein represents an important regulator of C/N homoeostasis. As shown previously for *S. elongatus* PCC 7942 [23], the absence of PII resulted in marked changes in the levels of many metabolites. However, metabolites of the primary C metabolism, which are known to be regulated by LC-shift conditions [11,12], were not so much affected in cells of Δ*glnB*. The lesser effects of C-specific metabolites correlated well with the almost unchanged expression of genes for the primary C metabolism as well as CCM components in LC-shifted cells of the *S. elongatus* PCC 7942 PII mutant [23]. Thus, we assume that another regulator is responsible for C-specific changes instead of PII.

However, the levels and pattern of metabolites associated with N assimilation were drastically affected in the *Synechocystis* PII mutant. Usually, shifts to LC induce a coordinated down-regulation of the N assimilation. Our results clearly indicate that this coordinated down-regulation of the N assimilation under LC conditions is not functioning in the PII mutant at the metabolic level. Many metabolites of the N metabolism such as ornithine, arginine, beta-alanine, and aspartate showed a pattern that gives a clear indication for N/C imbalance. Significantly decreased levels of 2OG and increased levels of citrate and isocitrate but not of arginine were also reported for the mutant defective the transcriptional regulator cyAbrB [41]. These authors proposed that the metabolic changes in this mutant might be due to a low expression of *glnB* in the Δ*cyabrB2* mutant, which is a direct target of cyAbrB. It is interesting to note that a deregulation of the LC-induced genes for CCM components also have been reported to occur in cells of the Δ*cyabrB2* mutant [42]. Future studies will reveal if this regulatory protein is somehow connected with PII to regulate specifically the C assimilation as it was shown for the regulator NtcA responsible for the PII-dependent specific regulation of N-assimilatory genes.

## Supplementary Files

Supplementary File 1 Supplementary XLSX (XLSX, 240 KB)
